# Age–period–cohort analysis of pulmonary tuberculosis reported incidence, China, 2006–2020

**DOI:** 10.1186/s40249-022-01009-4

**Published:** 2022-07-28

**Authors:** Zhe Dong, Qi-Qi Wang, Shi-Cheng Yu, Fei Huang, Jian-Jun Liu, Hong-Yan Yao, Yan-Lin Zhao

**Affiliations:** Chinese Center for Disease Control and Prevention, Beijing, China

**Keywords:** Pulmonary tuberculosis, Reported incidence, Age–period–cohort model, China

## Abstract

**Background:**

Tuberculosis (TB) poses a severe public health challenge in China and worldwide. This study evaluated the effects of age, period, and birth cohort on reported incidence trends of TB based on population and refined the characteristics of high-risk groups.

**Methods:**

Aggregate data that reported pulmonary tuberculosis (PTB) cases from China Tuberculosis Management Information System (TBIMS) from 2006 to 2020 were used to analyze effect coefficients through the age–period–cohort (APC) model based on intrinsic estimator (IE) method, and converted them into relative risk (*RR*) to estimate trends.

**Results:**

A total of 14.82 million cases of PTB were reported in China from 2006 to 2020, showing a continuous downward trend. The reporting rate increased with age by age group, with 70–74 years old being 2–3 times higher than that in 20–24 years old. APC analysis model showed that age effects were bimodal in 20–24 years old [*RR* = 2.29, 95% confidence interval (*CI*): 1.73–3.03] and 70–74 years old (*RR* = 1.95, 95% *CI:* 1.67–2.27), and lower than the overall average in the groups under 15 years old. Stratified results showed that the risk was higher for women under age 40 than men and higher for men over 40. The risk was higher in urban than in rural areas under 30 years old and slightly higher in rural than in urban between 30 and 64 years old. The risk for 15–34 years old was significantly higher in the east than in other regions. The period effects showed a decreasing trend, and the risk was higher in rural in recent years. Except for cohorts born in 1961–1965 and 2001–2005, where the *RR* increased, the later the cohort was born, the lower the risk. The cohort 1926–1930 in eastern had the highest risk (*RR* = 3.49, 95% *CI:* 2.44–4.98).

**Conclusions:**

The reported incidence of PTB continued to decline in China from 2006 to 2020. The young (20–24 years old) and the elderly (70–74 years old) were equally at high risk. There were differences in the age, period and cohort effects on PTB incidence among gender, urban–rural and regions. Our findings better reflected the characteristics of high-risk populations, thus contributing to the development of timely and effective intervention strategies, and providing clues for etiological research.

**Graphical abstract:**

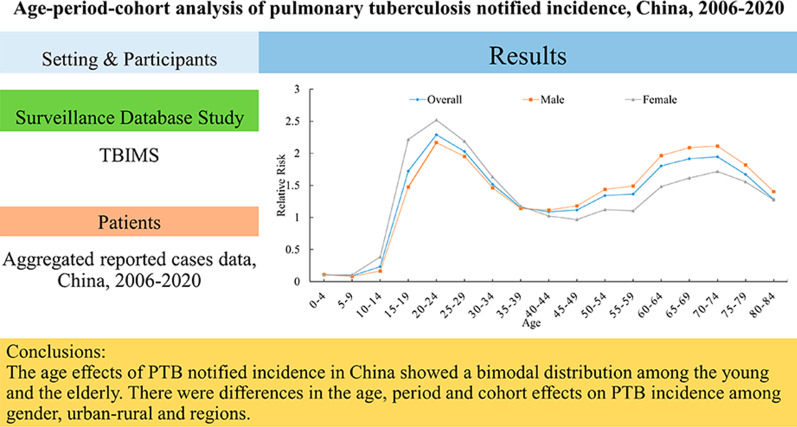

**Supplementary Information:**

The online version contains supplementary material available at 10.1186/s40249-022-01009-4.

## Background

Tuberculosis (TB) is a chronic infectious disease caused by *Mycobacterium tuberculosis* infection, also belongs to consumptive disease, mainly infecting the lungs, called pulmonary tuberculosis (PTB). About 5% of infected people will develop TB in the short term, about 95% will enter a long-term latent infection (LTBI) state, and about 5% of LTBI will progress to active patient years to decades later [1]. TB is the infectious disease that causes the most deaths by a single pathogen. By 2020, China was the world’s second-largest country with a high TB burden, and the decline rate slowed down in recent years, but still facing major public health challenges [2]. Understanding disease risk factors and identifying high-risk groups were significant for TB control and prevention strategies.

The onset and progression of TB result from a struggle between *M. tuberculosis* and the body’s immune defenses; and are influenced by various factors, including malnutrition, HIV/TB comorbidities, smoking, air pollution, etc. Age and gender may both biologically and socially affect their susceptibility. The reported incidence of TB in China’s elderly was 2 to 3 times that of young people [3], among males was higher than that in females [4]. There were also inconsistent results [5], and the actual effect of age needs further research. In addition, nutritional status in different birth cohorts might affect TB risk later in life and even in offspring [6]. A study in China found that prenatal and early life exposure to malnutrition in women who have experienced famine increases the risk of disease for themselves and their offspring [7], which should be investigated nationwide. The social economy was widely known as a critical factor in the TB epidemic, and the economy is highly unbalanced at regional levels in China. Therefore, it was necessary to compare the differences between regions.

Previous studies mainly described the epidemiological characteristics and trends of TB in China, but the analyses and exploration of the possible causes were still insufficient. Age–period–cohort (APC) is a quantitative analysis model often used in epidemiology and sociology [8, 9]. There were few studies on the application of APC in TB [7, 10–16], and only two at the national level in China [13, 16]. We collected PTB data reported to the China Tuberculosis Management Information System (TBIMS) in China, which has more comprehensive information and revised the onset date, but has not been used for such analysis. The current study aims to further assess the effects of age, period, and birth cohort on the reported incidence trend of PTB based on population among different gender, urban–rural and regions.

## Methods

### Data collection

The reported cases of PTB were derived from the TBIMS, a web-based database that was launched in 2005. TBIMS covers all TB control institutions and collects TB patients’ demographic, diagnostic, management, and outcome data [17]. This study used the aggregate annual case collection of each county in the mainland of China from 2006 to 2020, grouped by age and sex.

The annual aggregate data of the permanent resident population of county-level areas in the mainland of China from 2006 to 2020 were used and grouped by age and gender. The regional code includes an urban or rural division at each county level for each year, determining the rural–urban classification of tuberculosis cases and population data. These data were obtained from the Integrated Management Information System of the Chinese Center for Disease Prevention and Control. Standard population used in this study was the Chinese Population Census in 2010 [18].

### Statistical analysis

According to the National Bureau of Statistics, the 31 provincial-level administrative divisions (PLADs) in the mainland of China were divided into three regions (eastern, central, and western) based on factors such as geographical location and level of economic development [19]. The eastern region included Beijing, Fujian, Guangdong, Hainan, Hebei, Jiangsu, Liaoning, Shandong, Shanghai, Tianjin, and Zhejiang; The central region included Anhui, Heilongjiang, Henan, Hunan, Hubei, Jiangxi, Jilin, and Shanxi; The western region included Chongqing, Gansu, Guangxi, Guizhou, Inner Mongolia, Ningxia, Qinghai, Shaanxi, Sichuan, Tibet, Xinjiang, and Yunnan.

In view of changes in the underlying population, we used the method of direct standardization to calculate age-standardized rates (ASRs) to estimate the changes in nationwide PTB reporting rates over time.

In this study, APC was used to simultaneously evaluate the age, period, and cohort effects of trends in the reported incidence of PTB to disaggregate and explore the underlying physiological, social, historical, and environmental factors. Since there is a complete linear correlation between age, period, and cohort (cohort = period − age), the unique estimate of parameters cannot be obtained, also called a non-identification problem [20]. Based on the estimation function method, Fu proposed the intrinsic estimator (IE) based on the estimation function and matrix singular value decomposition [21]. The result is compelling and unique, with the advantages of no restriction assumption and a wide application range, which became one of the APC model research hotspots. Compared with the traditional generalized linear model, which assumes that two or more coefficients of a parameter vector are equal, the IE limits the geometric orientation of the parameter vector in the parameter space.

In this study, “age” referred to the biological age, and the age effect referred to the effects of different age groups on PTB. “Period” referred to the year in which cases were diagnosed, and period effect referred to external factors such as medical technology, policy interventions, and diagnostic methods on the PTB of all age groups within a specific period. “Birth cohort” referred to the birth year, and cohort effect referred to the effects of the social and economic environment, historical events, lifestyle and other factors on PTB since birth.

Before model analysis, the data had been aggregated to adapt model conditions with the same age, period, and cohort interval. The observation period included for as long as possible to show long-term trends. Thus, period, age and cohort were divided into three periods (2006–2010, 2011–2015, 2016–2020), 17 groups (0–4, 5–9, 10–14, …, 80–84) and 19 birth cohorts (1926–1930, …, 2016–2020), respectively with intervals of 5 years. Because neighbouring birth cohorts partially overlap, usually indicated by their middle year. For individuals aged 75–79 and 80–84 years from 2006 to 2010, their birth cohorts were from 1927 to 1935 and from 1922 to 1930. They are denoted as from 1931 to 1935 and from 1926 to 1930. Assuming that the count of PTB incidence obeys Poisson distribution, the APC model can be written as [22]:

$$\mathrm{log}\left({R}_{rijk}\right)=\mathrm{log}\left({E}_{i\dot{j}}/{P}_{i\dot{j}}\right)=\mu +\alpha \times {Age}_{i}+\beta \times {Period}_{j}+\gamma \times {Cohort}_{\kappa }, i=1,\dots ,\mathrm{a }j=1,\dots ,\mathrm{p}, \kappa =\mathrm{a}-i+j$$

where ${R}_{rijk}$ stands for the rate of different groups, ${E}_{i\dot{j}}$ and ${P}_{i\dot{j}}$ stand for the expected count of reported cases and the count of the exposed population, among the age group $i$ at the period $j$, respectively. α, β and γ represent the regression coefficients of the APC model’s age, period, and cohort effects, respectively. μ represents the overall mean effect. The model parameters (α, β, γ) were converted exponentially to represent the relative risk (*RR*) of a particular age, period, and birth cohort relative to each average level. (*RR* > 1 indicates higher risk relative to the average, and *RR* < 1 indicates lower risk relative to the average. For example, the *RR* of the male in cohort 1926–1930 was 3.48, indicating that the cohort was 3.48 times more likely to report incidence than the overall average of the male cohort.)

The analyses of this study were conducted in R software version 3.5.0 (The R Foundation for Statistical Computing, Vienna, Austria). The parameter estimation of APC-IE was performed using the APCG1 package.

## Results

### The overall trends in reported PTB incidence in China

**Fig. 1:**
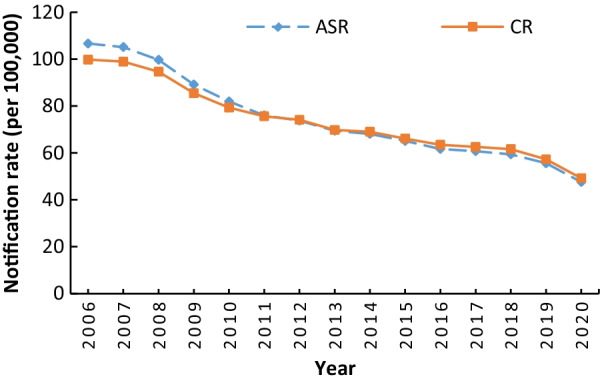
Trends in the reported incidence of pulmonary tuberculosis in China, 2006‒2020. *ASR* age-standardized rate, *CR* crude rate

A total of 14.82 million cases of PTB were reported in China from 2006 to 2020, with an average annual crude rate of 73.8 (/100,000) and an average annual ASR of 74.7 (/100,000). The reported incidence continued to decline over time, with a rapid decline from 2006 to 2010, a relatively stable decline from 2011 to 2018, and a significant decline from 2019 to 2020 (Fig. 1, Additional file 1: Table S1).

### The variation in age, period and cohort on reported PTB incidence

**Fig. 2:**
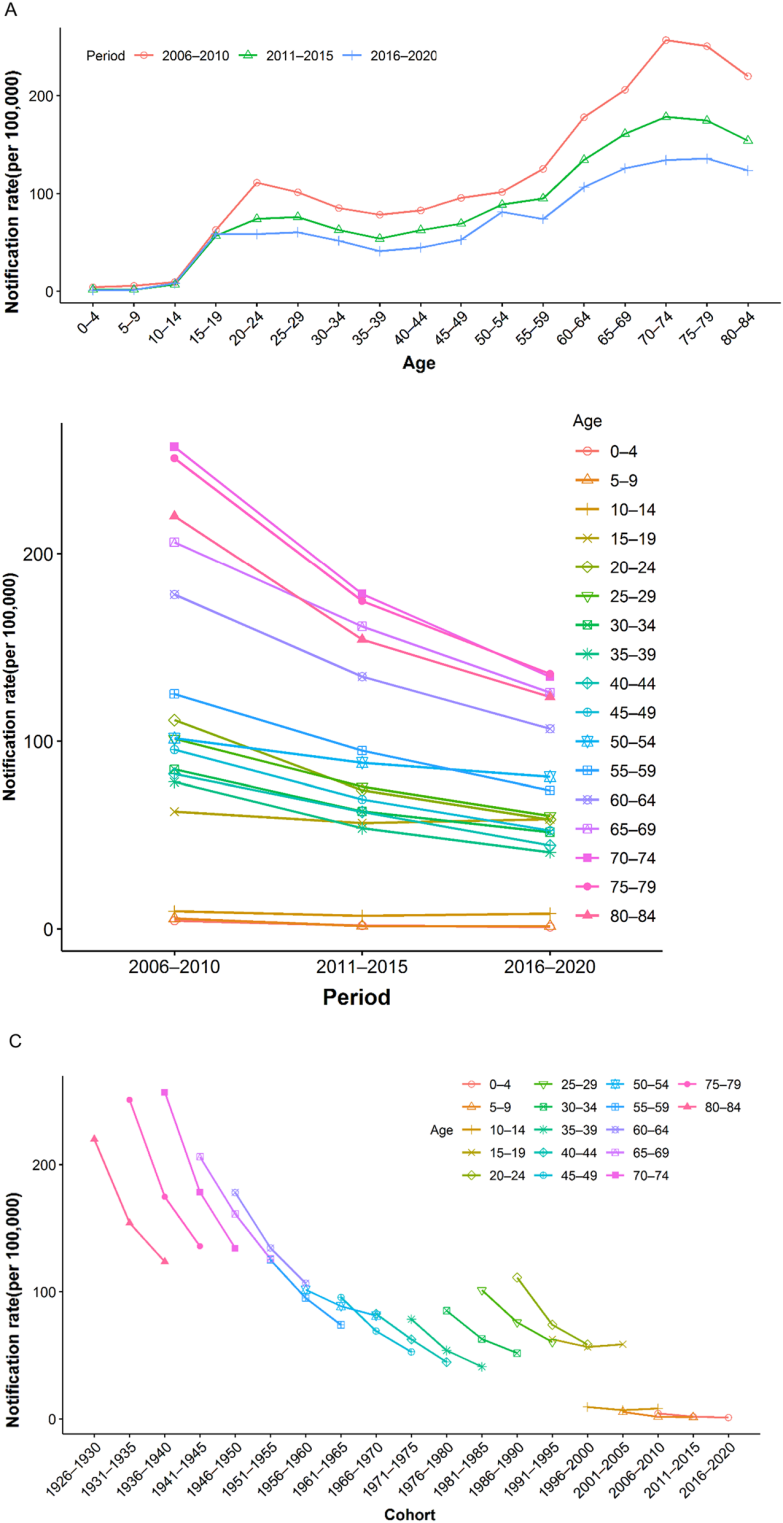
Variations in the reported incidence of pulmonary tuberculosis by **A** age, **B** period, and **C** birth cohort in the mainland of China, 2006‒2020

Figure 2 showed changes in the reported incidence of PTB by age, period and, birth cohort from 2006 to 2020 (Additional file 1: Table S2). The age-specific rate showed an upward trend among the three periods in Fig. 2A. The 20–24 years group reached the first peak, then decreased to 35–39 years group and raised again, and the 70–74 years group arrived at the second peak, which was twice as high as the first peak. Among them, the rate of the 50–54 age group showed an increasing trend from 2011 to 2020.

Figure 2B shown a downward trend in rate of all age groups from 2006 to 2020. It was higher in the group over 60 years, but the decline was larger, while it was more stable in the lower age group.

As shown in Fig. 2C, the birth cohort fluctuated significantly. The rates of most age groups decreased with the birth cohort and a sharp downward in the early cohort. The rate of age group 50–54 born between 1961 and 1970 decreased slower than that of the adjacent cohort in the period 2011–2020, the cohort after 1996 was relatively stable, and the 15–19 age group born between 2001 and 2005 showed an upward trend in period 2016–2020. The influence of age and cohort on PTB incidence cannot be presented independently. Hence, the APC model has more appropriate for this study design.

### The age–period–cohort model analysis of reported PTB incidence

#### Age effect of reported PTB incidence

**Fig. 3:**
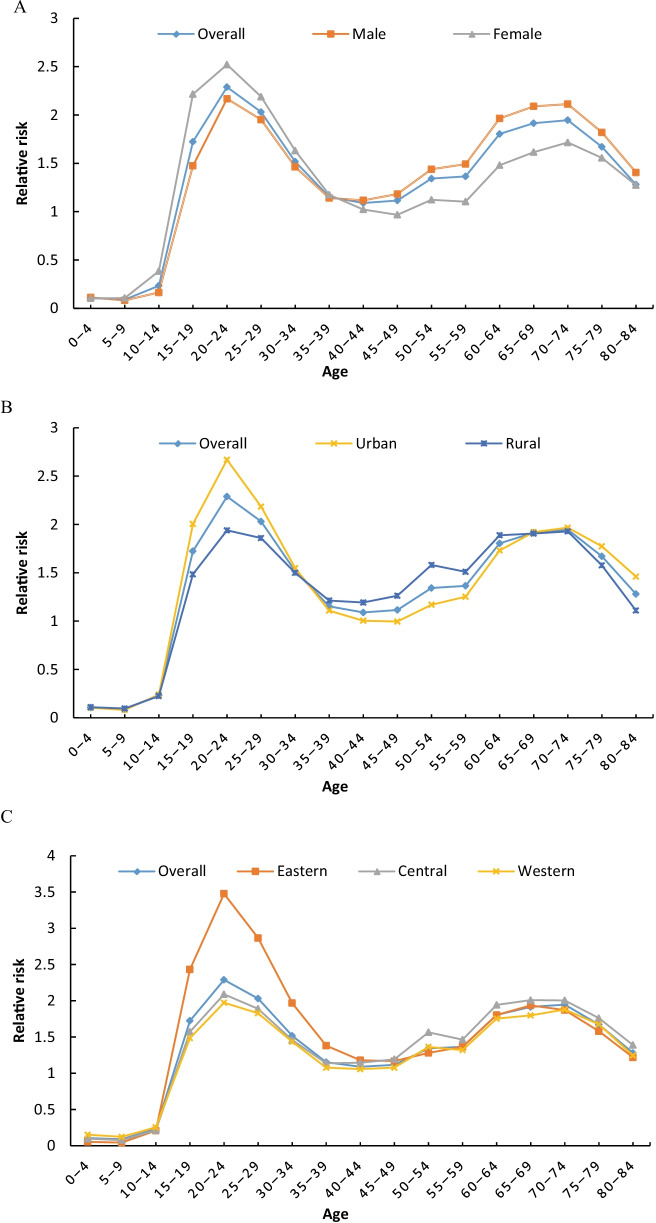
Age effects on the reported incidence of pulmonary tuberculosis in the mainland of China. **A** sex, **B** urban–rural, **C** Eastern, Central and Western

After controlling for period and cohort effects (Fig. 3, Additional file 1: Table S3), the age effect of reported PTB incidence in China showed a bimodal distribution with increasing age. The relative risk (*RR*) was slightly higher in 20–24 years (*RR* = 2.29, 95% *CI:* 1.73–3.03) than in 70–74 years (*RR* = 1.95, 95% *CI:* 1.67–2.27), the groups under 15 years were lower than the average level of the whole.

When stratified by sex, the risk was slightly higher for women under age 40 and higher for men over 40 years. When stratified by urban and rural areas, the risk was higher in urban than in rural for groups under 30 years, slightly higher in rural for groups between 30 and 64 years, and higher in urban for groups over 65 years. At the first peak, the risk of 20–24 years old in urban (*RR* = 2.67, 95% *CI:* 1.99–3.58) was higher than that in rural (*RR* = 1.94, 95% *CI:* 1.48–2.54). When stratified by regions, the risk was significantly higher in the eastern in the 15–39 years old, and highest in the 20–24 years old (*RR* = 3.48, 95% *CI:* 2.32–5.20). In the over 50 s, the risk was slightly higher in the central.

#### Period effect of reported PTB incidence

**Fig. 4:**
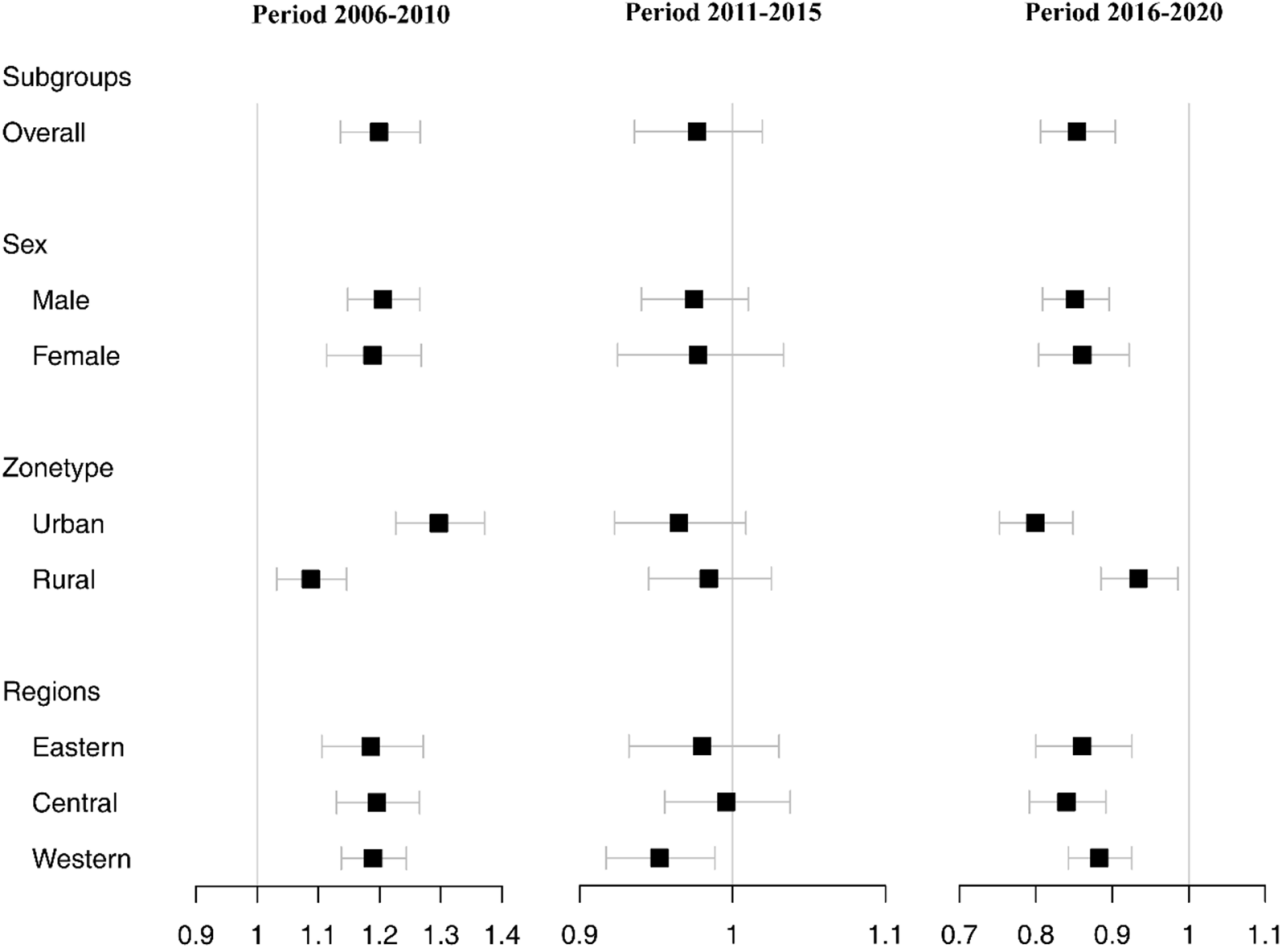
Period effects of reported incidence of pulmonary tuberculosis in the mainland of China during the three study periods. The “black dot” represents the relative risk (*RR*) values. The vertical line represents the invalid line whose *RR* value is 1. The “horizontal line” represents 95% *CI* of *RR* value, and crossing the “vertical line” indicates no statistical difference

The period effect decreased slowly from 2006 to 2020, with the highest risk in 2006–2010 (*RR* = 1.20, 95% *CI:* 1.14–1.27) and close to the overall average in period 2011–2015 (Fig. 4, Additional file 1: Table S3). After stratification, the period effects in different gender and regions were similar to those overall. It was noteworthy that the effect was slightly higher in urban (*RR* = 1.30, 95% *CI:* 1.23–1.37) than in rural (*RR* = 1.09, 95% *CI:* 1.03–1.15) between 2006 and 2010. However, the risk was highest in rural during 2016 to 2020 (*RR* = 0.93, 95% *CI:* 0.89–0.99).

#### Cohort effect of reported PTB incidence

**Fig. 5:**
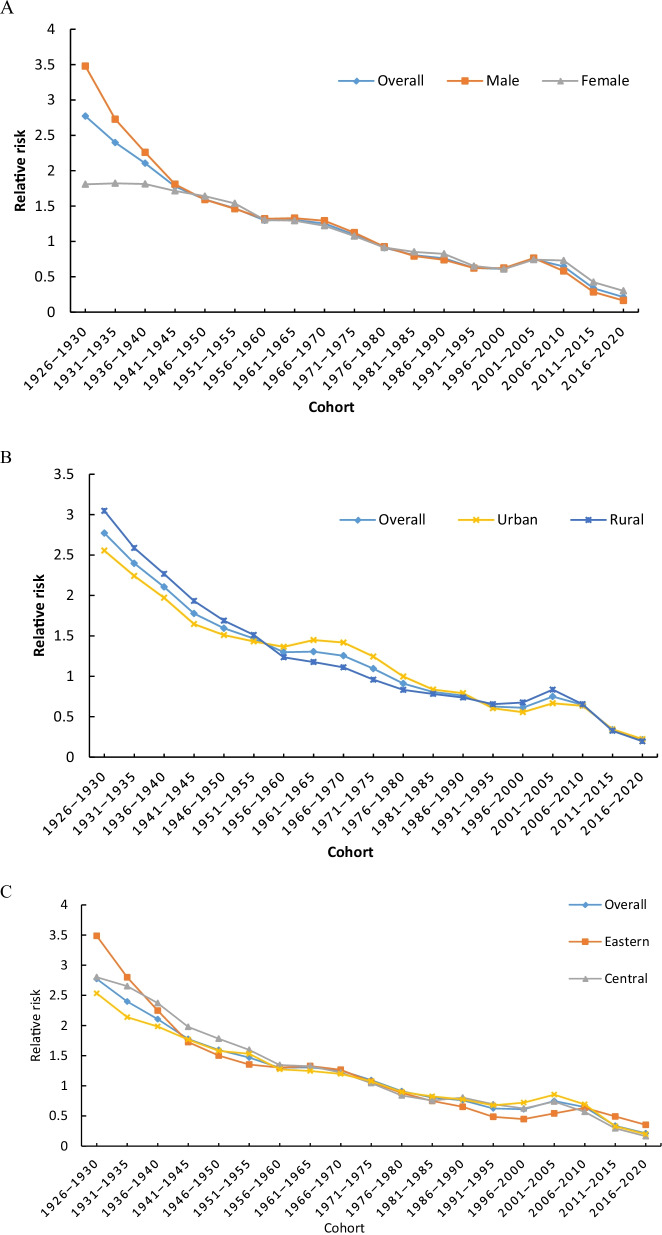
Cohort effects of reported incidence of pulmonary tuberculosis in the mainland of China. **A** sex, **B** urban–rural, **C** eastern, central and western

Cohort effects showed (Fig. 5, Additional file 1: Table S3) that the later the cohort was born, the lower the risk. Cohort 1926–1930 had the highest risk (*RR* = 2.77, 95% *CI:* 2.10–3.66), and the cohorts after 1976 were lower than the overall average. Cohorts 1961–1965 and 2001–2005 were at increased risk.

The risk was higher for men in the pre-1941 cohorts and higher for women in the post-2006 cohorts. Cohorts born before 1950 had a higher risk in rural, while cohorts for 1961–1980 had a higher risk in urban, and cohorts for 2001–2005 had the highest risk in rural. Before 1940, the risk was higher in the eastern and central, especially the cohort 1926–1930 (*RR* = 3.49, 95% *CI:* 2.44–4.98). The cohort effect declined faster in eastern, but the risk was slightly higher than in other regions after 2006.

## Discussions

The reported incidence of PTB in China continued to decline from 2006 to 2020. In the risk of PTB, the age effects presented a bimodal distribution, the period and cohort effects showed downward trends. Mover, the period effects declined slowly in recent years, and the birth cohort effects fluctuated significantly. There were significant differences in the effects changes after further stratification by gender, urban–rural and regions. The period effect after logarithmic transformation was relatively stable, implying that age was a more critical determinant.

According to the single-factor descriptive analysis, the reported rate of groups over 60 years was much higher than that of young people (2–3 times), consistent with previous reports [3, 23]. However, after controlling for period and cohort effects, the risk was similar in young and older people. Consistent with Wang’s conclusion [16], it was even slightly higher in younger adults. It also had come to similar conclusions in Argentina and India [13]. These results suggested that the APC model could better reflect the age effect pattern. We found that the risk was significantly lower under 15 years, possibly due to positive control effects of the neonatal BCG vaccination program. On the other hand, the rate of missed diagnosis among children was also high [24]. The risk was higher in all groups over 15 years old than the overall average, possibly due to cumulative exposure to *M. tuberculosis* infection, air pollution, smoking, and other determinants with age. The high risk of young people might be mainly due to continued transmission within the community, such as clustering in schools and frequent social activities, which increase the risk of exposure. The high risk of the elderly might be mainly due to the weakened immunity of the body, diabetes comorbidity, and other factors, which might cause the onset through the recent infection, or the reactivation of latent infection [25]. Therefore, the risk of PTB in both young and elderly in China was equally high. It was necessary to strengthen the case detection, treatment in specific age groups and develop new vaccines [26, 27].

The differences in age effect patterns between men and women might be due to complex interactions among biological, social, cultural, and economic factors [28, 29]. We found a slightly higher risk for women under 40 years and a higher risk for men over age 40. Brazil has also found that infection rates among young girls increase with age, while men begin to increase after puberty [30]. First, the behavioural shift in social contact during adolescence potentially drives the emergence of sex differences in TB epidemiology in adults [31]. Secondly, animal experiments have shown that estrogen protects against *M. tuberculosis* infection, reducing susceptibility. In pre-teen girls, estrogen fails to dominate, leading to increased risk [32]. Testosterone plays an immunosuppressive and harmful role in the pathogenesis of TB. Adult males are more likely to develop active disease due to smoking, biological or comorbidities [33], leading to higher mortality [34].

The age effect patterns might also differ between countries or regions due to demographic, socioeconomic, and cultural factors. In Hong Kong, China [14], the risk was unimodal in age 20–24, whereas in the United States [10], it was bimodal in children and young adults. We found that the risk of young people in urban was higher than that in rural areas, and the risk in eastern China was significantly higher than that in other regions, which was consistent with previous reports [35]. The broader social contact of younger in urban and eastern regions might increase the risk of infection. Furthermore, the school’s control strategy improved the ability of patient detection. The higher risk in rural among middle-aged people might be due to more unhealthy lifestyles. In addition, many peasant workers migrated to the cities in their youth and returned to the countryside in their middle and old age, which might be at the peak of the development of latent infection into active disease. Therefore, we found a noticeable increase in the rural population aged 50–54. A survey found that more than two-thirds of infections among migrants occurred after they moved to cities [36]. These reminded us of the need to tailor TB control strategies for high-risk groups in different regions.

The period effects of PTB incidence continued to decline, consistent with previous results [13]. This study found that the higher risk during the earlier period might be due to improved diagnostic techniques resulting from the directly observed treatment short-course (DOTS) roll-out, thus increasing case detection rates. The subsequent decline might be due to implementing a series of TB control strategies and the improvement of social, economic, and medical conditions. However, after 2011, the decline became smaller, indicating that we should pay more attention to innovative research. We found that the risk decreased significantly in urban, while the risk was higher in rural areas in recent years. The long-term control effect was significant in urban, mainly due to better DOTS promotion and health resource allocation from the early stage. Rural areas might have improved their capacity to detect cases in recent years due to improved sanitation.

Groups born in different generations might have different cohort effects due to the interaction of biological factors, demographic changes, intergenerational effects, and exposure to social events [37]. The risk was highest in the early cohort in China (especially in eastern), and the later the birth, the lower the risk, consistent with previous studies [16]. It was mainly due to the early experience of social unrest, frequent wars, economic depression, and other social events. War not only deteriorates the living environment and sanitary conditions but also poses a direct threat to life and health. In addition, a more extended period of cumulative exposure to environmental pollution might also be responsible [38]. After 1949, TB control efforts in China led to a decline in TB risk. The risk decline for the birth cohort 1950s was more significant than in recent years, possibly due to vaccination strategies adopted during that period. Since the reform and opening up and socialist modernization in 1978, the cohort effect has been lower than the average level of the whole. It was worth noting that the risk increased in the cohort 1961–1965. Mainly because China experienced its worst famine since the founding of the People’s Republic of China in the period, and malnutrition is a known risk factor for TB. We partially validated Cheng’s conclusion that nutritional status may influence TB risk later in life and even in offspring [7]. Because we further found that the decline in notifying rates in the cohort 1961–1970 at age 50–54 stage (period 2011–2020) slowed significantly. Moreover, the rate of decline in risk in the cohort 1981–1990 was slower, suggesting that intergenerational effects were possible. In addition, we found an increase in the cohort 2001–2005 (age 10–20). Firstly, due to DOTS implementation in 13 PLADs since 1992 and nationwide coverage in 2005, the case detection capacity has been improved significantly. Secondly, the intergenerational effect of parents in the 1980s might have increased the risk of family infection. Finally, increasing population mobility in the 1990s and early 2000s was also a possible reason.

This study also has several limitations. First, since only the last 15 years of data were available, we could not estimate earlier periods and cohort effects. Second, the number of PTB patients would be underestimated based on the reported incidence. Third, we evaluated the TB effects based on the groups, which may differ from the conclusion at the individual level. Ecological fallacies should be aware. Furthermore, this study did not include the factor analysis with covariates, calling for further research in the future.

## Conclusions

The age effects of PTB reported incidence in China showed a bimodal distribution, with both young and elderly having equally high risk. The period effects declined, while smaller decreases in recent years. The cohort effects declined, but there were notable fluctuations. War, malnutrition, and population migration might cause long-term effects on different birth cohorts. Our findings better reflected the characteristics of high-risk populations, thus contributing to the development of timely and effective intervention strategies, and providing clues for etiological research.

## Supplementary Information

**Additional file 1: Table S1.** The reported incidence of PTB in China, 2006–2020. **Table S2.** Variations in the reported incidence of PTB by age, period, and birth cohort in mainland China, 2006–2020. **Table S3-1.** APC-IE model parameters and relative risk of notified incidence of pulmonary tuberculosis by gender in China, 2006–2020. **Table S3-2.** APC-IE model parameters and relative risks of notified incidence of pulmonary tuberculosis in different regions of China, 2006–2020.

## Data Availability

The datasets used and analyzed during this study are available from the corresponding author on reasonable request.

## Abbreviations

APC: Age–period–cohort
ASRs: Age-standardized rates
*CI*: Confidence interval
DOTS: Directly observed treatment short-course
LTBI: Long-term latent infection
IE: Intrinsic estimator
PLADs: Provincial-level administrative divisions
PTB: Pulmonary tuberculosis
*RR*: Relative risk
TB: Tuberculosis
TBIMS: China Tuberculosis Management Information System
