# Supplementary material for: Age–period–cohort analysis of pulmonary tuberculosis reported incidence, China, 2006–2020
Source: Infect Dis Poverty. 2022 Jul 28;11:85. doi: 10.1186/s40249-022-01009-4 (PMC9331155; doi:10.1186/s40249-022-01009-4)
Supplement: Supplementary file 1 — Additional file 1: Table S1. The reported incidence of PTB in China, 2006–2020. Table S2. Variations in the reported incidence of PTB by age, period, and birth cohort in mainland China, 2006–2020. Table S3-1. APC-IE model parameters and relative risk of notified incidence of pulmonary tuberculosis by gender in China, 2006–2020. Table S3-2. APC-IE model parameters and relative risks of notified incidence of pulmonary tuberculosis in different regions of China, 2006–2020. [file 40249_2022_1009_MOESM1_ESM.docx]

**Additional file**

Table S1 The reported incidence (per 100,000) of pulmonary tuberculosis in China, 2006‒2020

| Year | ASR | CR |
| --- | --- | --- |
| 2006 | 106.64 | 99.78 |
| 2007 | 105.12 | 98.92 |
| 2008 | 99.69 | 94.62 |
| 2009 | 89.14 | 85.45 |
| 2010 | 81.94 | 79.34 |
| 2011 | 75.97 | 75.62 |
| 2012 | 73.76 | 74.10 |
| 2013 | 69.43 | 69.77 |
| 2014 | 68.08 | 69.02 |
| 2015 | 65.10 | 66.11 |
| 2016 | 61.67 | 63.47 |
| 2017 | 60.74 | 62.58 |
| 2018 | 59.38 | 61.60 |
| 2019 | 55.57 | 57.25 |
| 2020 | 47.58 | 49.21 |

Abbreviations: PTB = pulmonary tuberculosis; ASR = age-standardized rate; CR = crude rate

Table S2 Variations in the reported incidence of pulmonary tuberculosis by age, period, and birth cohort in the mainland of China, 2006‒2020

| Age | Period | Cohort | Count | Population | Incidence |
| --- | --- | --- | --- | --- | --- |
| 0‒4 | 2006‒2010 | 2006‒2010 | 17,730 | 412,247,179 | 4.30 |
| 0‒4 | 2011‒2015 | 2011‒2015 | 7,010 | 391,574,506 | 1.79 |
| 0‒4 | 2016‒2020 | 2016‒2020 | 4,144 | 407,564,393 | 1.02 |
| 5‒9 | 2006‒2010 | 2001‒2005 | 20,596 | 374,214,253 | 5.50 |
| 5‒9 | 2011‒2015 | 2006‒2010 | 6,160 | 388,288,295 | 1.59 |
| 5‒9 | 2016‒2020 | 2011‒2015 | 5,654 | 403,098,529 | 1.40 |
| 10‒14 | 2006‒2010 | 1996‒2000 | 42,768 | 455,197,688 | 9.40 |
| 10‒14 | 2011‒2015 | 2001‒2005 | 23,883 | 345,097,999 | 6.92 |
| 10‒14 | 2016‒2020 | 2006‒2010 | 28,504 | 349,285,391 | 8.16 |
| 15‒19 | 2006‒2010 | 1991‒1995 | 379,325 | 605,656,873 | 62.63 |
| 15‒19 | 2011‒2015 | 1996‒2000 | 261,234 | 461,646,827 | 56.59 |
| 15‒19 | 2016‒2020 | 2001‒2005 | 230,568 | 392,863,364 | 58.69 |
| 20‒24 | 2006‒2010 | 1986‒1990 | 622,434 | 559,705,511 | 111.21 |
| 20‒24 | 2011‒2015 | 1991‒1995 | 480,861 | 649,508,680 | 74.03 |
| 20‒24 | 2016‒2020 | 1996‒2000 | 328,199 | 561,503,281 | 58.45 |
| 25‒29 | 2006‒2010 | 1981‒1985 | 460,141 | 454,136,900 | 101.32 |
| 25‒29 | 2011‒2015 | 1986‒1990 | 394,251 | 519,264,391 | 75.92 |
| 25‒29 | 2016‒2020 | 1991‒1995 | 328,205 | 543,737,894 | 60.36 |
| 30‒34 | 2006‒2010 | 1976‒1980 | 426,305 | 500,875,454 | 85.11 |
| 30‒34 | 2011‒2015 | 1981‒1985 | 285,153 | 454,317,208 | 62.77 |
| 30‒34 | 2016‒2020 | 1986‒1990 | 257,645 | 498,079,629 | 51.73 |
| 35‒39 | 2006‒2010 | 1971‒1975 | 477,092 | 609,001,678 | 78.34 |
| 35‒39 | 2011‒2015 | 1976‒1980 | 301,704 | 560,522,859 | 53.83 |
| 35‒39 | 2016‒2020 | 1981‒1985 | 212,695 | 518,404,317 | 41.03 |
| 40‒44 | 2006‒2010 | 1966‒1970 | 485,874 | 587,663,214 | 82.68 |
| 40‒44 | 2011‒2015 | 1971‒1975 | 379,652 | 607,031,569 | 62.54 |
| 40‒44 | 2016‒2020 | 1976‒1980 | 248,557 | 556,103,038 | 44.70 |
| 45‒49 | 2006‒2010 | 1961‒1965 | 399,543 | 418,064,421 | 95.57 |
| 45‒49 | 2011‒2015 | 1966‒1970 | 418,074 | 604,728,033 | 69.13 |
| 45‒49 | 2016‒2020 | 1971‒1975 | 339,009 | 644,147,262 | 52.63 |
| 50‒54 | 2006‒2010 | 1956‒1960 | 477,539 | 470,073,107 | 101.59 |
| 50‒54 | 2011‒2015 | 1961‒1965 | 361,887 | 408,288,922 | 88.64 |
| 50‒54 | 2016‒2020 | 1966‒1970 | 384,226 | 473,541,933 | 81.14 |
| 55‒59 | 2006‒2010 | 1951‒1955 | 478,290 | 382,303,493 | 125.11 |
| 55‒59 | 2011‒2015 | 1956‒1960 | 415,652 | 437,435,076 | 95.02 |
| 55‒59 | 2016‒2020 | 1961‒1965 | 326,451 | 441,771,189 | 73.90 |
| 60‒64 | 2006‒2010 | 1946‒1950 | 449,064 | 252,206,094 | 178.05 |
| 60‒64 | 2011‒2015 | 1951‒1955 | 426,056 | 317,068,780 | 134.37 |
| 60‒64 | 2016‒2020 | 1956‒1960 | 385,178 | 361,175,176 | 106.65 |
| 65‒69 | 2006‒2010 | 1941‒1945 | 402,427 | 195,279,741 | 206.08 |
| 65‒69 | 2011‒2015 | 1946‒1950 | 345,296 | 214,351,845 | 161.09 |
| 65‒69 | 2016‒2020 | 1951‒1955 | 365,802 | 290,659,889 | 125.85 |
| 70‒74 | 2006‒2010 | 1936‒1940 | 401,009 | 156,104,862 | 256.88 |
| 70‒74 | 2011‒2015 | 1941‒1945 | 297,704 | 166,912,380 | 178.36 |
| 70‒74 | 2016‒2020 | 1946‒1950 | 274,425 | 204,436,577 | 134.23 |
| 75‒79 | 2006‒2010 | 1931‒1935 | 259,271 | 103,384,307 | 250.78 |
| 75‒79 | 2011‒2015 | 1936‒1940 | 220,096 | 126,055,935 | 174.60 |
| 75‒79 | 2016‒2020 | 1941‒1945 | 202,059 | 148,855,170 | 135.74 |
| 80‒84 | 2006‒2010 | 1926‒1930 | 117,682 | 53,503,082 | 219.95 |
| 80‒84 | 2011‒2015 | 1931‒1935 | 110,317 | 71,600,025 | 154.07 |
| 80‒84 | 2016‒2020 | 1936‒1940 | 114,525 | 92,736,440 | 123.50 |

Table S3-1. APC-IE model parameters and relative risk of notified incidence of pulmonary tuberculosis by gender in China, 2006‒2020

| Factor | Overall | | |  | Male | | |  | Female | | |
| --- | --- | --- | --- | --- | --- | --- | --- | --- | --- | --- | --- |
|  | Coefficient (95% *CI*) | *RR* (95% *CI*) | *P*-value |  | Coefficient (95% *CI*) | *RR* (95% *CI*) | *P*-value |  | Coefficient (95% *CI*) | *RR* (95% *CI*) | *P*-value |
| **Age（years）** | |  |  |  |  |  |  |  |  |  |  |
| 0‒4 | ‒2.24 (‒3.09, ‒1.4) | 0.11 (0.05‒0.25) | 0.0000 |  | ‒2.18 (‒2.98, ‒1.39) | 0.11 (0.05‒0.25) | 0.0000 |  | ‒2.26 (‒3.2, ‒1.32) | 0.1 (0.04‒0.27) | 0.0000 |
| 5‒9 | ‒2.41 (‒3.04, ‒1.78) | 0.09 (0.05‒0.17) | 0.0000 |  | ‒2.5 (‒3.08, ‒1.91) | 0.08 (0.05‒0.15) | 0.0000 |  | ‒2.24 (‒2.94, ‒1.54) | 0.11 (0.05‒0.21) | 0.0000 |
| 10‒14 | ‒1.45 (‒1.87, ‒1.04) | 0.23 (0.15‒0.35) | 0.0000 |  | ‒1.81 (‒2.23, ‒1.39) | 0.16 (0.11‒0.25) | 0.0000 |  | ‒0.96 (‒1.37, ‒0.54) | 0.38 (0.25‒0.58) | 0.0000 |
| 15‒19 | 0.54 (0.25, 0.84) | 1.72 (1.28‒2.32) | 0.0000 |  | 0.39 (0.1, 0.67) | 1.47 (1.11‒1.96) | 0.0080 |  | 0.8 (0.47, 1.12) | 2.22 (1.61‒3.05) | 0.0000 |
| 20‒24 | 0.83 (0.55, 1.11) | 2.29 (1.73‒3.03) | 0.0000 |  | 0.77 (0.51, 1.04) | 2.17 (1.66‒2.83) | 0.0000 |  | 0.92 (0.62, 1.23) | 2.52 (1.85‒3.43) | 0.0000 |
| 25‒29 | 0.71 (0.44, 0.98) | 2.03 (1.55‒2.66) | 0.0000 |  | 0.67 (0.42, 0.92) | 1.95 (1.52‒2.51) | 0.0000 |  | 0.78 (0.48, 1.08) | 2.19 (1.62‒2.95) | 0.0000 |
| 30‒34 | 0.42 (0.16, 0.68) | 1.52 (1.17‒1.97) | 0.0020 |  | 0.38 (0.14, 0.62) | 1.46 (1.15‒1.86) | 0.0020 |  | 0.49 (0.19, 0.79) | 1.63 (1.21‒2.2) | 0.0010 |
| 35‒39 | 0.14 (‒0.11, 0.39) | 1.15 (0.9‒1.48) | 0.2710 |  | 0.13 (‒0.1, 0.36) | 1.14 (0.91‒1.43) | 0.2550 |  | 0.16 (‒0.14, 0.46) | 1.17 (0.87‒1.58) | 0.2920 |
| 40‒44 | 0.09 (‒0.15, 0.32) | 1.09 (0.86‒1.38) | 0.4740 |  | 0.11 (‒0.1, 0.32) | 1.12 (0.91‒1.37) | 0.2910 |  | 0.02 (‒0.27, 0.31) | 1.02 (0.77‒1.37) | 0.8790 |
| 45‒49 | 0.11 (‒0.1, 0.32) | 1.12 (0.9‒1.38) | 0.3150 |  | 0.17 (‒0.02, 0.35) | 1.18 (0.98‒1.42) | 0.0750 |  | ‒0.03 (‒0.31, 0.24) | 0.97 (0.74‒1.27) | 0.8120 |
| 50‒54 | 0.29 (0.11, 0.48) | 1.34 (1.12‒1.62) | 0.0020 |  | 0.36 (0.21, 0.52) | 1.44 (1.23‒1.68) | 0.0000 |  | 0.12 (‒0.13, 0.36) | 1.12 (0.88‒1.43) | 0.3490 |
| 55‒59 | 0.31 (0.15, 0.47) | 1.36 (1.16‒1.61) | 0.0000 |  | 0.4 (0.26, 0.54) | 1.49 (1.3‒1.71) | 0.0000 |  | 0.1 (‒0.12, 0.32) | 1.1 (0.89‒1.37) | 0.3710 |
| 60‒64 | 0.59 (0.45, 0.73) | 1.8 (1.57‒2.08) | 0.0000 |  | 0.68 (0.55, 0.8) | 1.96 (1.74‒2.22) | 0.0000 |  | 0.39 (0.21, 0.58) | 1.48 (1.23‒1.78) | 0.0000 |
| 65‒69 | 0.65 (0.51, 0.79) | 1.92 (1.67‒2.2) | 0.0000 |  | 0.74 (0.61, 0.86) | 2.09 (1.85‒2.36) | 0.0000 |  | 0.48 (0.3, 0.66) | 1.62 (1.35‒1.93) | 0.0000 |
| 70‒74 | 0.67 (0.51, 0.82) | 1.95 (1.67‒2.27) | 0.0000 |  | 0.75 (0.61, 0.89) | 2.11 (1.84‒2.43) | 0.0000 |  | 0.54 (0.35, 0.73) | 1.72 (1.42‒2.07) | 0.0000 |
| 75‒79 | 0.51 (0.32, 0.7) | 1.67 (1.38‒2.02) | 0.0000 |  | 0.6 (0.43, 0.77) | 1.82 (1.53‒2.17) | 0.0000 |  | 0.44 (0.22, 0.67) | 1.56 (1.24‒1.95) | 0.0000 |
| 80‒84 | 0.25 (0.01, 0.49) | 1.28 (1.01‒1.63) | 0.0430 |  | 0.34 (0.12, 0.56) | 1.41 (1.13‒1.75) | 0.0020 |  | 0.24 (‒0.04, 0.53) | 1.27 (0.96‒1.69) | 0.0950 |
| **Period** |  |  |  |  |  |  |  |  |  |  |  |
| 2006‒2010 | 0.18 (0.13, 0.24) | 1.2 (1.14‒1.27) | 0.0000 |  | 0.19 (0.14, 0.24) | 1.21 (1.15‒1.27) | 0.0000 |  | 0.17 (0.11, 0.24) | 1.19 (1.11‒1.27) | 0.0000 |
| 2011‒2015 | ‒0.02 (‒0.07, 0.02) | 0.98 (0.94‒1.02) | 0.2800 |  | ‒0.03 (‒0.06, 0.01) | 0.97 (0.94‒1.01) | 0.1610 |  | ‒0.02 (‒0.08, 0.03) | 0.98 (0.92‒1.03) | 0.4220 |
| 2016‒2020 | ‒0.16 (‒0.22, ‒0.1) | 0.85 (0.81‒0.9) | 0.0000 |  | ‒0.16 (‒0.21, ‒0.11) | 0.85 (0.81‒0.9) | 0.0000 |  | ‒0.15 (‒0.22, ‒0.08) | 0.86 (0.8‒0.92) | 0.0000 |
| **Cohort** |  |  |  |  |  |  |  |  |  |  |  |
| 1926‒1930 | 1.02 (0.74, 1.3) | 2.77 (2.1‒3.66) | 0.0000 |  | 1.25 (1, 1.49) | 3.48 (2.72‒4.44) | 0.0000 |  | 0.59 (0.24, 0.94) | 1.81 (1.28‒2.56) | 0.0010 |
| 1931‒1935 | 0.87 (0.66, 1.09) | 2.4 (1.93‒2.98) | 0.0000 |  | 1 (0.81, 1.2) | 2.73 (2.24‒3.32) | 0.0000 |  | 0.6 (0.34, 0.86) | 1.82 (1.4‒2.37) | 0.0000 |
| 1936‒1940 | 0.74 (0.57, 0.92) | 2.11 (1.76‒2.51) | 0.0000 |  | 0.81 (0.65, 0.98) | 2.26 (1.92‒2.65) | 0.0000 |  | 0.59 (0.38, 0.81) | 1.81 (1.47‒2.24) | 0.0000 |
| 1941‒1945 | 0.57 (0.42, 0.73) | 1.78 (1.52‒2.08) | 0.0000 |  | 0.59 (0.45, 0.73) | 1.81 (1.57‒2.08) | 0.0000 |  | 0.54 (0.35, 0.73) | 1.71 (1.42‒2.07) | 0.0000 |
| 1946‒1950 | 0.47 (0.31, 0.62) | 1.59 (1.36‒1.86) | 0.0000 |  | 0.46 (0.33, 0.6) | 1.59 (1.38‒1.83) | 0.0000 |  | 0.49 (0.3, 0.69) | 1.64 (1.35‒1.99) | 0.0000 |
| 1951‒1955 | 0.38 (0.21, 0.56) | 1.47 (1.24‒1.74) | 0.0000 |  | 0.38 (0.23, 0.53) | 1.46 (1.25‒1.7) | 0.0000 |  | 0.43 (0.22, 0.64) | 1.54 (1.24‒1.91) | 0.0000 |
| 1956‒1960 | 0.26 (0.06, 0.46) | 1.3 (1.06‒1.58) | 0.0100 |  | 0.28 (0.1, 0.45) | 1.32 (1.11‒1.57) | 0.0020 |  | 0.27 (0.02, 0.51) | 1.3 (1.02‒1.67) | 0.0360 |
| 1961‒1965 | 0.27 (0.04, 0.49) | 1.3 (1.04‒1.64) | 0.0230 |  | 0.28 (0.08, 0.49) | 1.33 (1.09‒1.63) | 0.0060 |  | 0.26 (‒0.03, 0.54) | 1.29 (0.97‒1.72) | 0.0750 |
| 1966‒1970 | 0.23 (‒0.03, 0.48) | 1.25 (0.97‒1.62) | 0.0820 |  | 0.26 (0.03, 0.49) | 1.29 (1.03‒1.62) | 0.0280 |  | 0.2 (‒0.11, 0.51) | 1.22 (0.9‒1.66) | 0.2050 |
| 1971‒1975 | 0.09 (‒0.19, 0.37) | 1.09 (0.83‒1.45) | 0.5300 |  | 0.12 (‒0.14, 0.37) | 1.12 (0.87‒1.45) | 0.3710 |  | 0.07 (‒0.26, 0.4) | 1.07 (0.77‒1.5) | 0.6690 |
| 1976‒1980 | ‒0.09 (‒0.39, 0.2) | 0.91 (0.68‒1.23) | 0.5380 |  | ‒0.08 (‒0.35, 0.2) | 0.92 (0.7‒1.22) | 0.5760 |  | ‒0.09 (‒0.43, 0.25) | 0.91 (0.65‒1.29) | 0.6060 |
| 1981‒1985 | ‒0.22 (‒0.53, 0.09) | 0.81 (0.59‒1.1) | 0.1690 |  | ‒0.23 (‒0.52, 0.06) | 0.79 (0.59‒1.06) | 0.1170 |  | ‒0.16 (‒0.5, 0.18) | 0.85 (0.6‒1.2) | 0.3590 |
| 1986‒1990 | ‒0.27 (‒0.59, 0.04) | 0.76 (0.55‒1.05) | 0.0920 |  | ‒0.3 (‒0.61, 0) | 0.74 (0.55‒1) | 0.0510 |  | ‒0.19 (‒0.54, 0.15) | 0.82 (0.58‒1.16) | 0.2720 |
| 1991‒1995 | ‒0.47 (‒0.8, ‒0.14) | 0.62 (0.45‒0.87) | 0.0060 |  | ‒0.47 (‒0.8, ‒0.15) | 0.62 (0.45‒0.86) | 0.0040 |  | ‒0.43 (‒0.79, ‒0.07) | 0.65 (0.46‒0.93) | 0.0180 |
| 1996‒2000 | ‒0.49 (‒0.85, ‒0.13) | 0.61 (0.43‒0.88) | 0.0070 |  | ‒0.47 (‒0.82, ‒0.12) | 0.62 (0.44‒0.88) | 0.0080 |  | ‒0.5 (‒0.88, ‒0.12) | 0.61 (0.41‒0.89) | 0.0110 |
| 2001‒2005 | ‒0.29 (‒0.68, 0.1) | 0.75 (0.51‒1.11) | 0.1470 |  | ‒0.27 (‒0.65, 0.11) | 0.76 (0.52‒1.11) | 0.1610 |  | ‒0.3 (‒0.71, 0.11) | 0.74 (0.49‒1.12) | 0.1570 |
| 2006‒2010 | ‒0.44 (‒1, 0.12) | 0.65 (0.37‒1.13) | 0.1260 |  | ‒0.54 (‒1.1, 0.02) | 0.58 (0.33‒1.02) | 0.0590 |  | ‒0.32 (‒0.88, 0.25) | 0.73 (0.42‒1.28) | 0.2720 |
| 2011‒2015 | ‒1.09 (‒2.16, ‒0.01) | 0.34 (0.11‒0.99) | 0.0480 |  | ‒1.26 (‒2.28, ‒0.23) | 0.28 (0.1‒0.79) | 0.0160 |  | ‒0.86 (‒2.02, 0.3) | 0.42 (0.13‒1.35) | 0.1450 |
| 2016‒2020 | ‒1.55 (‒3.55, 0.46) | 0.21 (0.03‒1.58) | 0.1300 |  | ‒1.8 (‒3.72, 0.11) | 0.16 (0.02‒1.12) | 0.0650 |  | ‒1.2 (‒3.33, 0.92) | 0.3 (0.04‒2.52) | 0.2680 |

Table S3-2. APC-IE model parameters and relative risks of notified incidence of pulmonary tuberculosis in different regions of China, 2006‒2020

| Factor | Urban | | |  | Rural | | |  | Eastern | |  |  | Central | | |  | Western | | |
| --- | --- | --- | --- | --- | --- | --- | --- | --- | --- | --- | --- | --- | --- | --- | --- | --- | --- | --- | --- |
|  | Coefficient (95% *CI*) | *RR* (95% *CI*) | *P*-value |  | Coefficient (95% *CI*) | *RR* (95% *CI*) | *P*-value |  | Coefficient (95% *CI*) | *RR* (95% *CI*) | *P-*value |  | Coefficient (95% *CI*) | *RR* (95% *CI*) | *P*-value |  | Coefficient (95% *CI*) | *RR* (95% *CI*) | *P*-value |
| **Age（years）** | |  |  |  |  |  |  |  |  |  |  |  |  |  |  |  |  |  |  |
| 0‒4 | ‒2.27 (‒3.15, ‒1.38) | 0.1 (0.04‒0.25) | 0.0000 |  | ‒2.21 (‒3.03, ‒1.39) | 0.11 (0.05‒0.25) | 0.0000 |  | ‒2.92 (‒4.23, ‒1.61) | 0.05 (0.01‒0.2) | 0.000 |  | ‒2.33 (‒3.29, ‒1.37) | 0.1 (0.04‒0.25) | 0.000 |  | ‒1.88 (‒2.49, ‒1.28) | 0.15 (0.08‒0.28) | 0.000 |
| 5‒9 | ‒2.51 (‒3.21, ‒1.81) | 0.08 (0.04‒0.16) | 0.0000 |  | ‒2.35 (‒2.94, ‒1.76) | 0.1 (0.05‒0.17) | 0.0000 |  | ‒3.11 (‒4.21, ‒2) | 0.04 (0.01‒0.13) | 0.000 |  | ‒2.56 (‒3.27, ‒1.84) | 0.08 (0.04‒0.16) | 0.000 |  | ‒2.1 (‒2.54, ‒1.65) | 0.12 (0.08‒0.19) | 0.000 |
| 10‒14 | ‒1.43 (‒1.87, ‒0.99) | 0.24 (0.15‒0.37) | 0.0000 |  | ‒1.49 (‒1.89, ‒1.1) | 0.22 (0.15‒0.33) | 0.0000 |  | ‒1.57 (‒2.17, ‒0.97) | 0.21 (0.11‒0.38) | 0.000 |  | ‒1.54 (‒1.99, ‒1.09) | 0.21 (0.14‒0.34) | 0.000 |  | ‒1.37 (‒1.68, ‒1.05) | 0.25 (0.19‒0.35) | 0.000 |
| 15‒19 | 0.7 (0.38, 1.01) | 2.01 (1.46‒2.75) | 0.0000 |  | 0.4 (0.11, 0.68) | 1.48 (1.12‒1.98) | 0.0070 |  | 0.89 (0.45, 1.33) | 2.43 (1.57‒3.78) | 0.000 |  | 0.45 (0.13, 0.78) | 1.57 (1.13‒2.18) | 0.007 |  | 0.4 (0.17, 0.62) | 1.48 (1.18‒1.87) | 0.001 |
| 20‒24 | 0.98 (0.69, 1.28) | 2.67 (1.99‒3.58) | 0.0000 |  | 0.66 (0.39, 0.93) | 1.94 (1.48‒2.54) | 0.0000 |  | 1.25 (0.84, 1.65) | 3.48 (2.32‒5.2) | 0.000 |  | 0.74 (0.43, 1.04) | 2.09 (1.54‒2.84) | 0.000 |  | 0.68 (0.46, 0.9) | 1.97 (1.58‒2.46) | 0.000 |
| 25‒29 | 0.78 (0.5, 1.06) | 2.18 (1.65‒2.89) | 0.0000 |  | 0.62 (0.36, 0.88) | 1.86 (1.43‒2.41) | 0.0000 |  | 1.05 (0.68, 1.42) | 2.87 (1.98‒4.15) | 0.000 |  | 0.64 (0.35, 0.93) | 1.89 (1.41‒2.52) | 0.000 |  | 0.6 (0.39, 0.82) | 1.83 (1.48‒2.26) | 0.000 |
| 30‒34 | 0.44 (0.17, 0.71) | 1.55 (1.18‒2.02) | 0.0010 |  | 0.4 (0.15, 0.66) | 1.5 (1.16‒1.93) | 0.0020 |  | 0.68 (0.33, 1.02) | 1.97 (1.39‒2.78) | 0.000 |  | 0.38 (0.1, 0.65) | 1.46 (1.1‒1.93) | 0.008 |  | 0.36 (0.15, 0.57) | 1.43 (1.16‒1.77) | 0.001 |
| 35‒39 | 0.1 (‒0.16, 0.36) | 1.11 (0.85‒1.44) | 0.4370 |  | 0.19 (‒0.05, 0.44) | 1.21 (0.95‒1.55) | 0.1230 |  | 0.32 (0, 0.65) | 1.38 (1‒1.91) | 0.050 |  | 0.13 (‒0.14, 0.4) | 1.14 (0.87‒1.49) | 0.340 |  | 0.07 (‒0.14, 0.28) | 1.08 (0.87‒1.33) | 0.486 |
| 40‒44 | 0 (‒0.24, 0.25) | 1 (0.79‒1.28) | 0.9730 |  | 0.18 (‒0.05, 0.4) | 1.19 (0.95‒1.5) | 0.1280 |  | 0.17 (‒0.13, 0.46) | 1.18 (0.88‒1.58) | 0.270 |  | 0.13 (‒0.11, 0.38) | 1.14 (0.9‒1.46) | 0.278 |  | 0.06 (‒0.14, 0.25) | 1.06 (0.87‒1.29) | 0.579 |
| 45‒49 | 0 (‒0.22, 0.22) | 1 (0.8‒1.24) | 0.9690 |  | 0.23 (0.03, 0.44) | 1.26 (1.03‒1.55) | 0.0260 |  | 0.15 (‒0.11, 0.42) | 1.17 (0.9‒1.52) | 0.246 |  | 0.18 (‒0.04, 0.39) | 1.19 (0.96‒1.48) | 0.111 |  | 0.07 (‒0.11, 0.26) | 1.08 (0.9‒1.29) | 0.424 |
| 50‒54 | 0.16 (‒0.04, 0.35) | 1.17 (0.96‒1.42) | 0.1120 |  | 0.46 (0.28, 0.63) | 1.58 (1.32‒1.89) | 0.0000 |  | 0.25 (0.02, 0.47) | 1.28 (1.02‒1.61) | 0.033 |  | 0.45 (0.26, 0.63) | 1.56 (1.3‒1.88) | 0.000 |  | 0.31 (0.15, 0.47) | 1.36 (1.16‒1.6) | 0.000 |
| 55‒59 | 0.23 (0.05, 0.4) | 1.25 (1.06‒1.49) | 0.0100 |  | 0.41 (0.26, 0.57) | 1.51 (1.29‒1.76) | 0.0000 |  | 0.31 (0.11, 0.51) | 1.37 (1.12‒1.66) | 0.002 |  | 0.38 (0.22, 0.54) | 1.46 (1.25‒1.72) | 0.000 |  | 0.28 (0.14, 0.42) | 1.32 (1.15‒1.52) | 0.000 |
| 60‒64 | 0.55 (0.4, 0.7) | 1.73 (1.49‒2.01) | 0.0000 |  | 0.64 (0.5, 0.77) | 1.89 (1.65‒2.16) | 0.0000 |  | 0.59 (0.42, 0.76) | 1.8 (1.52‒2.15) | 0.000 |  | 0.66 (0.52, 0.8) | 1.94 (1.69‒2.23) | 0.000 |  | 0.56 (0.44, 0.68) | 1.75 (1.56‒1.98) | 0.000 |
| 65‒69 | 0.65 (0.5, 0.8) | 1.92 (1.66‒2.23) | 0.0000 |  | 0.65 (0.51, 0.78) | 1.91 (1.67‒2.17) | 0.0000 |  | 0.66 (0.48, 0.84) | 1.94 (1.62‒2.31) | 0.000 |  | 0.7 (0.56, 0.84) | 2.01 (1.74‒2.31) | 0.000 |  | 0.59 (0.47, 0.7) | 1.8 (1.6‒2.02) | 0.000 |
| 70‒74 | 0.68 (0.51, 0.84) | 1.97 (1.67‒2.32) | 0.0000 |  | 0.66 (0.51, 0.8) | 1.93 (1.67‒2.23) | 0.0000 |  | 0.63 (0.43, 0.83) | 1.87 (1.53‒2.29) | 0.000 |  | 0.7 (0.53, 0.86) | 2 (1.7‒2.36) | 0.000 |  | 0.63 (0.5, 0.76) | 1.88 (1.66‒2.13) | 0.000 |
| 75‒79 | 0.57 (0.37, 0.77) | 1.77 (1.45‒2.17) | 0.0000 |  | 0.46 (0.28, 0.63) | 1.58 (1.32‒1.89) | 0.0000 |  | 0.46 (0.21, 0.71) | 1.58 (1.23‒2.02) | 0.000 |  | 0.57 (0.36, 0.77) | 1.76 (1.44‒2.15) | 0.000 |  | 0.52 (0.36, 0.67) | 1.67 (1.44‒1.95) | 0.000 |
| 80‒84 | 0.38 (0.13, 0.63) | 1.46 (1.14‒1.88) | 0.0030 |  | 0.1 (‒0.12, 0.33) | 1.11 (0.88‒1.39) | 0.3730 |  | 0.2 (‒0.11, 0.51) | 1.22 (0.89‒1.66) | 0.212 |  | 0.33 (0.08, 0.58) | 1.39 (1.08‒1.79) | 0.011 |  | 0.22 (0.02, 0.42) | 1.25 (1.03‒1.51) | 0.027 |
| **Period** |  |  |  |  |  |  |  |  |  |  |  |  |  |  |  |  |  |  |  |
| 2006‒2010 | 0.26 (0.2, 0.32) | 1.3 (1.23‒1.37) | 0.0000 |  | 0.08 (0.03, 0.14) | 1.09 (1.03‒1.15) | 0.0020 |  | 0.17 (0.1, 0.24) | 1.19 (1.11‒1.27) | 0.000 |  | 0.18 (0.12, 0.24) | 1.2 (1.13‒1.27) | 0.000 |  | 0.17 (0.13, 0.22) | 1.19 (1.14‒1.24) | 0.000 |
| 2011‒2015 | ‒0.04 (‒0.08, 0.01) | 0.96 (0.92‒1.01) | 0.1150 |  | ‒0.02 (‒0.06, 0.02) | 0.98 (0.95‒1.03) | 0.4500 |  | ‒0.02 (‒0.07, 0.03) | 0.98 (0.93‒1.03) | 0.431 |  | 0 (‒0.05, 0.04) | 1 (0.96‒1.04) | 0.842 |  | ‒0.05 (‒0.09, ‒0.01) | 0.95 (0.92‒0.99) | 0.010 |
| 2016‒2020 | ‒0.22 (‒0.28, ‒0.16) | 0.8 (0.75‒0.85) | 0.0000 |  | ‒0.07 (‒0.12, ‒0.01) | 0.93 (0.89‒0.99) | 0.0130 |  | ‒0.15 (‒0.22, ‒0.08) | 0.86 (0.8‒0.93) | 0.000 |  | ‒0.17 (‒0.23, ‒0.11) | 0.84 (0.79‒0.89) | 0.000 |  | ‒0.12 (‒0.17, ‒0.08) | 0.88 (0.84‒0.93) | 0.000 |
| **Cohort** |  |  |  |  |  |  |  |  |  |  |  |  |  |  |  |  |  |  |  |
| 1926‒1930 | 0.94 (0.65, 1.22) | 2.56 (1.92‒3.4) | 0.0000 |  | 1.11 (0.84, 1.39) | 3.05 (2.32‒4) | 0.0000 |  | 1.25 (0.89, 1.61) | 3.49 (2.44‒4.98) | 0.000 |  | 1.03 (0.74, 1.32) | 2.8 (2.09‒3.75) | 0.000 |  | 0.93 (0.7, 1.16) | 2.53 (2.01‒3.19) | 0.000 |
| 1931‒1935 | 0.81 (0.58, 1.03) | 2.24 (1.79‒2.81) | 0.0000 |  | 0.95 (0.74, 1.16) | 2.59 (2.1‒3.19) | 0.0000 |  | 1.03 (0.74, 1.31) | 2.8 (2.1‒3.72) | 0.000 |  | 0.98 (0.74, 1.21) | 2.65 (2.1‒3.34) | 0.000 |  | 0.76 (0.58, 0.94) | 2.14 (1.79‒2.55) | 0.000 |
| 1936‒1940 | 0.68 (0.49, 0.87) | 1.97 (1.64‒2.38) | 0.0000 |  | 0.82 (0.65, 0.99) | 2.27 (1.91‒2.69) | 0.0000 |  | 0.81 (0.58, 1.04) | 2.25 (1.78‒2.84) | 0.000 |  | 0.86 (0.67, 1.06) | 2.37 (1.96‒2.87) | 0.000 |  | 0.68 (0.54, 0.83) | 1.98 (1.72‒2.29) | 0.000 |
| 1941‒1945 | 0.5 (0.33, 0.67) | 1.65 (1.39‒1.95) | 0.0000 |  | 0.66 (0.51, 0.81) | 1.93 (1.66‒2.24) | 0.0000 |  | 0.55 (0.34, 0.75) | 1.72 (1.4‒2.12) | 0.000 |  | 0.68 (0.51, 0.85) | 1.98 (1.67‒2.35) | 0.000 |  | 0.57 (0.44, 0.7) | 1.77 (1.56‒2) | 0.000 |
| 1946‒1950 | 0.41 (0.25, 0.58) | 1.51 (1.28‒1.78) | 0.0000 |  | 0.52 (0.38, 0.67) | 1.69 (1.46‒1.96) | 0.0000 |  | 0.41 (0.21, 0.6) | 1.5 (1.23‒1.83) | 0.000 |  | 0.58 (0.41, 0.74) | 1.78 (1.51‒2.1) | 0.000 |  | 0.46 (0.33, 0.58) | 1.58 (1.39‒1.79) | 0.000 |
| 1951‒1955 | 0.36 (0.18, 0.54) | 1.43 (1.19‒1.72) | 0.0000 |  | 0.41 (0.25, 0.57) | 1.51 (1.29‒1.78) | 0.0000 |  | 0.3 (0.09, 0.52) | 1.35 (1.09‒1.68) | 0.006 |  | 0.47 (0.29, 0.65) | 1.6 (1.33‒1.92) | 0.000 |  | 0.43 (0.29, 0.57) | 1.53 (1.33‒1.76) | 0.000 |
| 1956‒1960 | 0.31 (0.1, 0.52) | 1.36 (1.11‒1.68) | 0.0040 |  | 0.21 (0.02, 0.4) | 1.24 (1.02‒1.49) | 0.0270 |  | 0.26 (0.02, 0.51) | 1.3 (1.02‒1.66) | 0.034 |  | 0.3 (0.09, 0.51) | 1.35 (1.09‒1.66) | 0.005 |  | 0.24 (0.08, 0.4) | 1.27 (1.08‒1.5) | 0.004 |
| 1961‒1965 | 0.37 (0.13, 0.61) | 1.45 (1.14‒1.84) | 0.0020 |  | 0.16 (‒0.05, 0.38) | 1.18 (0.95‒1.46) | 0.1420 |  | 0.28 (0, 0.57) | 1.33 (1‒1.76) | 0.049 |  | 0.28 (0.04, 0.52) | 1.32 (1.04‒1.69) | 0.022 |  | 0.22 (0.03, 0.41) | 1.25 (1.03‒1.5) | 0.022 |
| 1966‒1970 | 0.35 (0.08, 0.61) | 1.42 (1.09‒1.85) | 0.0100 |  | 0.1 (‒0.14, 0.35) | 1.11 (0.87‒1.42) | 0.3980 |  | 0.24 (‒0.08, 0.55) | 1.27 (0.92‒1.74) | 0.147 |  | 0.2 (‒0.07, 0.47) | 1.22 (0.93‒1.6) | 0.152 |  | 0.18 (‒0.03, 0.39) | 1.2 (0.97‒1.48) | 0.090 |
| 1971‒1975 | 0.22 (‒0.07, 0.51) | 1.24 (0.93‒1.66) | 0.1420 |  | ‒0.04 (‒0.31, 0.23) | 0.96 (0.73‒1.25) | 0.7550 |  | 0.06 (‒0.29, 0.42) | 1.07 (0.75‒1.52) | 0.725 |  | 0.04 (‒0.26, 0.35) | 1.05 (0.77‒1.42) | 0.775 |  | 0.07 (‒0.16, 0.3) | 1.08 (0.86‒1.35) | 0.532 |
| 1976‒1980 | 0 (‒0.31, 0.31) | 1 (0.73‒1.36) | 0.9840 |  | ‒0.18 (‒0.47, 0.1) | 0.83 (0.62‒1.11) | 0.2080 |  | ‒0.13 (‒0.51, 0.26) | 0.88 (0.6‒1.29) | 0.519 |  | ‒0.18 (‒0.5, 0.15) | 0.84 (0.61‒1.16) | 0.288 |  | ‒0.11 (‒0.35, 0.13) | 0.9 (0.71‒1.14) | 0.375 |
| 1981‒1985 | ‒0.18 (‒0.5, 0.14) | 0.84 (0.61‒1.15) | 0.2720 |  | ‒0.25 (‒0.54, 0.05) | 0.78 (0.58‒1.05) | 0.1040 |  | ‒0.29 (‒0.7, 0.12) | 0.75 (0.5‒1.12) | 0.163 |  | ‒0.28 (‒0.62, 0.06) | 0.76 (0.54‒1.06) | 0.110 |  | ‒0.19 (‒0.44, 0.05) | 0.82 (0.65‒1.05) | 0.123 |
| 1986‒1990 | ‒0.23 (‒0.57, 0.1) | 0.79 (0.57‒1.1) | 0.1670 |  | ‒0.3 (‒0.61, 0) | 0.74 (0.54‒1) | 0.0510 |  | ‒0.43 (‒0.86, 0) | 0.65 (0.42‒1) | 0.052 |  | ‒0.22 (‒0.57, 0.14) | 0.8 (0.57‒1.15) | 0.228 |  | ‒0.26 (‒0.51, ‒0.01) | 0.77 (0.6‒0.99) | 0.042 |
| 1991‒1995 | ‒0.5 (‒0.85, ‒0.15) | 0.6 (0.43‒0.86) | 0.0050 |  | ‒0.42 (‒0.74, ‒0.1) | 0.65 (0.48‒0.9) | 0.0090 |  | ‒0.72 (‒1.19, ‒0.26) | 0.49 (0.3‒0.77) | 0.002 |  | ‒0.36 (‒0.74, 0.01) | 0.69 (0.48‒1.01) | 0.055 |  | ‒0.39 (‒0.65, ‒0.14) | 0.68 (0.52‒0.87) | 0.003 |
| 1996‒2000 | ‒0.59 (‒0.97, ‒0.21) | 0.56 (0.38‒0.81) | 0.0030 |  | ‒0.39 (‒0.74, ‒0.05) | 0.68 (0.48‒0.95) | 0.0250 |  | ‒0.8 (‒1.31, ‒0.29) | 0.45 (0.27‒0.75) | 0.002 |  | ‒0.47 (‒0.87, ‒0.07) | 0.62 (0.42‒0.93) | 0.022 |  | ‒0.33 (‒0.6, ‒0.05) | 0.72 (0.55‒0.95) | 0.019 |
| 2001‒2005 | ‒0.41 (‒0.82, 0.01) | 0.67 (0.44‒1.01) | 0.0560 |  | ‒0.18 (‒0.55, 0.19) | 0.83 (0.58‒1.21) | 0.3350 |  | ‒0.61 (‒1.17, ‒0.05) | 0.54 (0.31‒0.95) | 0.033 |  | ‒0.3 (‒0.74, 0.13) | 0.74 (0.48‒1.14) | 0.173 |  | ‒0.16 (‒0.45, 0.14) | 0.85 (0.64‒1.14) | 0.290 |
| 2006‒2010 | ‒0.45 (‒1.05, 0.14) | 0.63 (0.35‒1.15) | 0.1350 |  | ‒0.42 (‒0.95, 0.1) | 0.65 (0.39‒1.11) | 0.1140 |  | ‒0.45 (‒1.25, 0.34) | 0.64 (0.29‒1.41) | 0.265 |  | ‒0.57 (‒1.2, 0.07) | 0.57 (0.3‒1.07) | 0.079 |  | ‒0.37 (‒0.78, 0.05) | 0.69 (0.46‒1.05) | 0.082 |
| 2011‒2015 | ‒1.07 (‒2.23, 0.09) | 0.34 (0.11‒1.09) | 0.0710 |  | ‒1.12 (‒2.13, ‒0.11) | 0.33 (0.12‒0.89) | 0.0290 |  | ‒0.71 (‒2.26, 0.84) | 0.49 (0.1‒2.33) | 0.371 |  | ‒1.23 (‒2.46, 0.01) | 0.29 (0.09‒1.01) | 0.052 |  | ‒1.11 (‒1.89, ‒0.34) | 0.33 (0.15‒0.71) | 0.005 |
| 2016‒2020 | ‒1.5 (‒3.62, 0.61) | 0.22 (0.03‒1.84) | 0.1640 |  | ‒1.63 (‒3.53, 0.27) | 0.2 (0.03‒1.3) | 0.0920 |  | ‒1.04 (‒3.71, 1.63) | 0.35 (0.02‒5.1) | 0.444 |  | ‒1.81 (‒4.23, 0.61) | 0.16 (0.01‒1.84) | 0.142 |  | ‒1.62 (‒3.06, ‒0.18) | 0.2 (0.05‒0.84) | 0.028 |
